# Targeting Glutamine Induces Apoptosis: A Cancer Therapy Approach

**DOI:** 10.3390/ijms160922830

**Published:** 2015-09-22

**Authors:** Lian Chen, Hengmin Cui

**Affiliations:** 1 Key Laboratory of Animal Diseases and Environmental Hazards of Sichuan Province, Sichuan Agriculture University, Ya’an 625014, China; E-Mail: lianchen87@163.com; 2 College of Veterinary Medicine, Sichuan Agricultural University, Ya’an 625014, China

**Keywords:** glutamine metabolism, apoptosis, glutaminase, cancer

## Abstract

Glutamine metabolism has been proved to be dysregulated in many cancer cells, and is essential for proliferation of most cancer cells, which makes glutamine an appealing target for cancer therapy. In order to be well used by cells, glutamine must be transported to cells by specific transporters and converted to glutamate by glutaminase. There are currently several drugs that target glutaminase under development or clinical trials. Also, glutamine metabolism restriction has been proved to be effective in inhibiting tumor growth both *in vivo* and *vitro* through inducing apoptosis, growth arrest and/or autophagy. Here, we review recent researches about glutamine metabolism in cancer, and cell death induced by targeting glutamine, and their potential roles in cancer therapy.

## 1. Introduction

Glutamine is the richest amino acid in the human body [1]. After being transported to cells, glutamine acts as precursor for the synthesis of many amino acids, proteins, nucleotides, and other biologically important molecules, and provides NADPH (nicotinamide adenine dinucleotide phosphate) and GSH (glutathione) to maintain redox homeostasis [2]. Thus, glutamine plays a critical role in cell growth and proliferation. In recent years, glutamine has come to the attention of researchers due to its increase and fast consumption in most cancers when compared to normal tissues. Glutamine metabolism restriction has been proved to be effective in suppressing cancer cell growth while glutamine supplementation can induce or inhibit cell death according to the cell type [3]. In any case, extracellular glutamine level affects the susceptibility of cancer cells to different apoptotic triggers. Glutamine deprivation has been reported to sensitize Hela cells to Fas (CD95) ligand, TNF-α (tumor necrosis factor-α) and heat shock-mediated apoptosis [4].

As the first critical enzyme for glutamine metabolism, GLS (glutaminase) has been reported to be positively correlated with malignancy in cancers and with growth rate in normal cells [5]. Specific inhibitors of GLS have been shown to induce cell death in cancer cells. Furthermore, some of them are currently in clinical trials, and more are at the bench. Interestingly, GLS activity is influenced by oncogenes, e.g., *MYC* and *p53*. This character connects glutamine metabolism with malignancy development, and highlights the possibility of targeting glutamine metabolism in cancer therapy. However, although recent numerous investigations have greatly expanded our understanding of glutamine metabolism in cancers and their potential value in the cancer therapy, there are drug resistance and adverse effects during lab and clinical researches. In this review, we discuss current knowledge of glutamine metabolism and how oncogenes regulate glutamine metabolism in cancers. We also address the GLS inhibitors and their utilization in the cancer therapy.

## 2. Glutamine Metabolism

### 2.1. Glutamine Metabolism in Normal Tissue

Glutamine circulates in the blood and is stored mainly in skeletal muscles, and also in other organs such as lung and brain [2]. Hepatocytes serve as a glutamine producer and consumer according to the body’s metabolic needs [2]. Besides, small intestine and kidney also use glutamine to maintain acid-base balance [6]. Glutamine is a nonessential amino acid, and is produced partly from amidation of glutamic acid by ammonia derived from purine metabolism and/or taken up from the circulation, partly from transamination and subsequent amidation of glucose-derived α-oxoglutarate [7].

**Figure 1:**
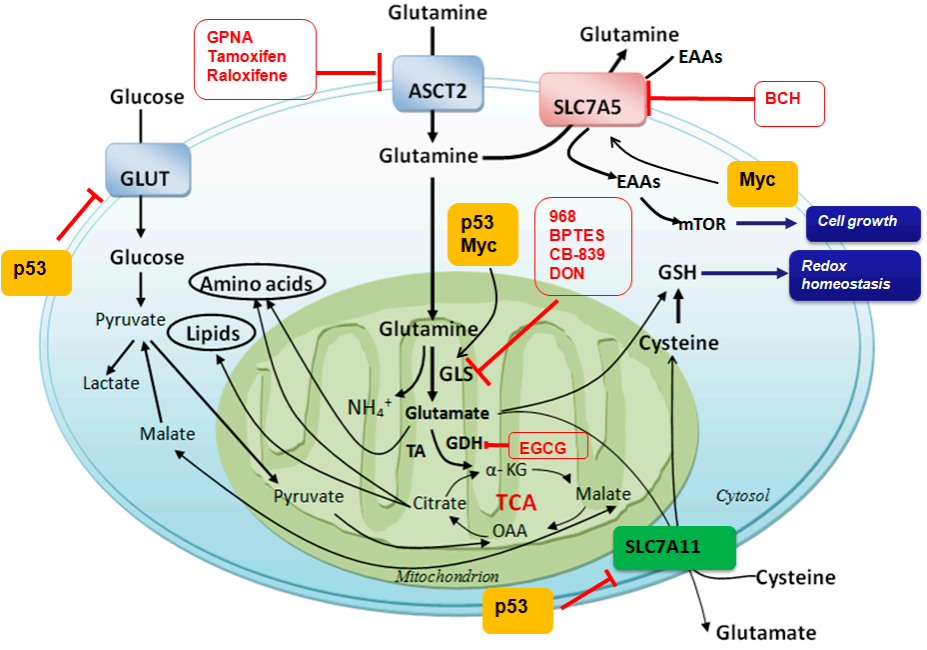
Glutamine metabolism and potent targets for cancer therapy. After transporting into cytosol by LAT1 (l-type amino acid transporters 1), ASCT2 (system ASC amino acid transporters 2) and other transporters, glutamine is catalyzed by glutaminase and converts to glutamate and ammonia. It then provides macromolecular material for ammonia acid and lipid syntheses. Glutamine is also used to exchange EAAs, which could activate mTOR and promote cell growth. Glutamate is also used to exchange extracellular cysteine for GSH production. GLS is a key enzyme for glutamine metabolism, which can be inhibited by several inhibitors including 968, BPTES and CB-839, accompanying with other inhibitors of glutamine metabolism are shown in red circle. GLS, glutaminase; GDH, glutamate dehydrogenase; TA, transaminase; OAA, oxaloacetate; BCH, 2-aminobicyclo-(2,2,1)-heptane-2-carboxylic acid; GPNA, γ-l-glutamylp-nitroanilide; EGCG, epigallocatechin gallate; EAAs, essential ammonia acids; mTOR, mammalian target of rapamycin; BPTES, bis-2-(5-phenylacetamido-1,2,4-thiadiazol-2-yl)ethyl sulfide 3; 968, 5-(3-bromo-4-(dimethylamino) phenyl)-2,2-dimethyl-2,3,5,6-tetrahydrobenzo[a]phenanthridin-4(1H)-one; CB-839, *N*-(5-(4-(6-((2-(3-(trifluoromethoxy)phenyl)acetyl)amino)-3-pyridazinyl)butyl)-1,3,4-thiadiazol-2-yl)-2-pyridineacetamide. ┴, inhibiting effect; bold black arrow, main metabolic pathway and transportation of glutamine; black arrow, metabolic pathways of glutamine and glucose.

Glutamine is first catalyzed by GLS to form glutamate and an ammonium ion. Glutamate is subsequently converted to α-KG (α-ketoglutarate) by glutamate dehydrogenase, and then α-KG enters TCA (tricarboxylic acid cycle) cycle to provide energy and macromolecular material sources (Figure 1). Particularly, glutamine metabolism provides carbon for OAA (oxaloacetic acid), acetyl-CoA and citrate production, lipogenesis, and nitrogen for purine, pyrimidine and DNA synthesis [8], and reductive power NADPH to support cell proliferation [9]. In addition to GLS, the donation of glutamine’s amido nitrogen to nucleotides or hexosamines mediated by glutamine: fructose-6-phosphate amidotransferase could contribute a fraction of the glutamate pool as well. This might become more prominent during the cell cycle stage characterized by transient increases in nucleotide biosynthesis or other activities [10]. It is noteworthy that immune cells require high levels of extracellular glutamine for proper activation. It has been reported that leukocytes metabolize glutamine at rates that are comparable to glucose utilization, and even higher in some instances [7].

### 2.2. Glutamine Metabolism in Cancers

**Figure 2:**
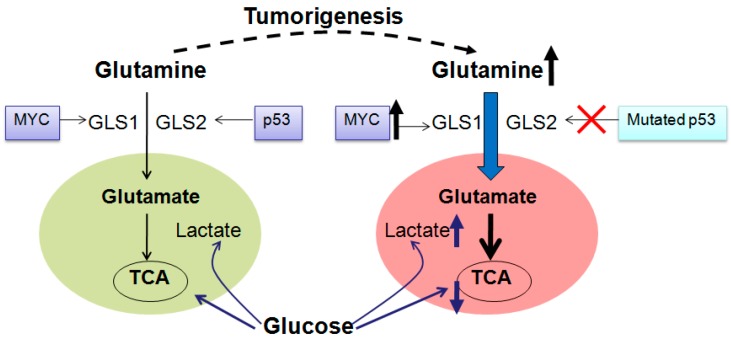
Glutamine consumption is increased in most tumors. During tumorigenesis, glucose derived lactate is increased, and at the same time, contribution of glucose to TCA is decreased. Accompanied with glucose metabolism change, glutamine metabolism is up-regulated to compensate energy and macromolecular for cell proliferation and growth. *p53* is mutated, while *MYC* is overexpressed, which promotes glutamine metabolism by upregulating GLS1 activity during tumorigenesis. GLS1 is highly expressed in many tumors and promotes tumor proliferation. In contrast, GLS2 expression is reduced in some tumors. GLS, glutaminase; TCA, tricarboxylic acid cycle. Bold arrow, increased glutamine metabolism, decreased glucose metabolism and mutated *MYC*; dashed line, tumorigenesis procedure.

It is well known that cancer cells utilize glucose in a dissipative way and use glucose to produce ATP (adenosine-triphosphate) through aerobic glycolysis regardless of oxygen availability (Warburg effect) [11]. To satisfy fast proliferation, cancer cells have to use another energy source, glutamine, which produces ATP through glutamine-driven oxidative phosphorylation [12]. Due to continual loss of citrate from the TCA cycle in proliferating cells, especially in cancer cells, replenishment of TCA intermediates (anaplerosis) is necessary, and glutamine consumption is increased (Figure 2). Higher consumption of glutamine in cancers is used for anabolic metabolism requirements, which produces metabolic building blocks, such as nucleic acids, lipids and proteins (Figure 1) [13], and increases GSH production for cell death resistance. Also, higher consumption of glutamine in cancers is used for EAAs (essential amino acids) exchanges that are required for cell growth and mTOR (mammalian target of rapamycin) activation, which then initiates protein translation and cell growth (Figure 1) [14]. Accompanied by increased glutamine consumption in most cancer cells, e.g., triple negative breast cancer cell MX-1 [15], neuroblastoma cell [16] and human myeloid cell KU812F, GLS activity is also much higher than normal cells. Elevated glutamine metabolism not only provides energy and substrates for cancer cells’ growth and proliferation, but also makes glutamine a potent candidate in the cancer therapy (Figure 1).

In order to be well used by cells, glutamine has to across the plasma membrane and mitochondrial inner membrane, and metabolizes in mitochondria [17], which is mediated by specific transporters. There are several different transport systems for amino acids to cross plasma membrane, e.g., Na^+^-dependent system A, Na^+^-dependent system N, Na^+^-dependent system ASC (alanine serine cystein) and Na^+^-independent system L [18]. Na^+^-dependent system A maintains elevated intracellular concentrations of non-essential amino acids like glutamine and alanine that can be exchanged to essential amino acids through Na^+^-dependent system ASC and Na^+^-independent system L. Na^+^-dependent system N can determine the extent of glutamine accumulation because it is able to mediate net glutamine efflux [19]. Glutamine transporters vary between normal and cancer cells. Glutamine transport in rat astrocytoma C6 cells and SK-N-SE cells is predominantly mediated by ASCT2 [20,21]. However, in normal rat astrocytes *in situ*, glutamine transport is mediated by a Na^+^-dependent system N, SN1 [20]. During transformation, ASCT2 and LAT1 are the two main transporters for glutamine uptake [22,23,24]. It has been hypothesized that LAT1 provides the essential amino acids to enhance cancer cell growth via mTOR-stimulated translation, and that ASCT2 maintains the cytoplasmic amino acid pool to drive LAT1 function [18]. This cooperation of ASCT2 and LAT1 suppresses apoptosis, and fuels the energy economy via net delivery of glutamine [18].

However, not all cancers are addicted to glutamine. There are some cancer cells that can survive and proliferate by relying on glucose without glutamine. Cheng *et al.* have uncovered a compensatory relationship between GLS1 and PC (pyruvate carboxylase). Glucose-derived pyruvate serves as an anaplerotic precursor, and thus, cells rely more on glucose in GLS1 silencing condition. PC activity is induced in low-glutamine condition and is required for cells to escape glutamine addiction [25]. This compensatory relationship also needs to be taken into consideration in developing new cancer therapy that targets glutamine or glucose metabolism.

### 2.3. Regulation of Glutamine Metabolism in Cancers

In cancers, glutamine metabolism is highly regulated by several factors, such as *MYC*, *p53*, Ras and HIF (hypoxia-inducible factor). This modulation is maintaining cancer growth, and also is one of the reasons for carcinogenesis.

#### 2.3.1. *MYC* in Glutamine Metabolism in Cancers

The proto-oncogene *MYC* regulates about 15% of genes in genomes from flies to humans [26]. It includes *N-MYC*, *C-MYC* and *L-MYC* those are deregulated, mutated or amplified in most human tumors [27,28,29]. They can be activated by mitogenic signals and drive cell proliferation. *C-MYC* is broadly deregulated in many human cancers, *N-MYC* expression is more restricted to neural cancers and *L-MYC* is predominantly found in small cell lung cancer [16].

In some cancers, *MYC* amplification is involved in glutamine addiction (Figure 2) [30]. Glutamine addiction is correlated to *MYC*-induced redirection of glucose carbon away from mitochondria that is a result of LDHA (lactate dehydrogenase) activation [14]. More specifically, *MYC*-induction leads to the diversion of glucose-derived pyruvate away from mitochondria and is converted to lactate [30]. As a result, *MYC*-transformed cells become dependent on glutamine anapleurosis for maintaining the mitochondrial integrity and TCA cycle function. Also, *MYC* is likely to increase activities of GLS1 [31] (as shown in the next part) and glutamine synthetase [32]. In addition, *MYC* probably binds to the promoter elements of glutamine transporters, which is associated with enhanced levels of glutamine transporters, e.g., SLC7A5 (solute carrier family 7 member 5, LAT1) and ASCT2 (Figure 1) [30,31]. *N-MYC* overexpression stimulates mRNA and protein expression of the catalytic subunit of GCL (glutamate-cysteine ligase), and causes rate-limiting step in GSH biosynthesis, which increases GSH level and provides resistance to oxidative damage [33]. Therefore, targeting *MYC* can provide a therapeutic window for cancers that have *MYC* amplification. Down-regulation of *N-MYC* expression has been proved to induce apoptosis, and to decrease proliferation and/or neuronal differentiation in neuroblastoma cells *in vitro* [34]. Similar results are also observed in lymphoma, leukemia, osteosarcoma, hepatocellular carcinoma, squamous carcinoma, and pancreatic carcinoma [35].

However, as many other strategies, targeting *MYC* is also a double-edged sword, and does not always promote cancer therapy under some circumstances. Switching off *N-MYC* suppresses the caspase-3 process and PARP (poly(ADP-ribose) polymerase) cleavages in TET21N cells treated with cisplatin [36]. Besides, transfection of *N-MYC* in *N-MYC* single copy SK-N-SH and NIH3T3 cells can promote DMAP1 (Dnmt1 associated protein) expression, which induces apoptosis via *p53* activation [37].

#### 2.3.2. *p53* in Glutamine Metabolism in Cancers

As one of the cell fate determinants, *p53* gene is found to be mutated or dismantled in most human cancers (Figure 2) [38]. It is widely accepted that *p53* is a tumor suppressor gene, which is able to induce cell cycle arrest and apoptosis under DNA damage, hypoxia or oncogene activation conditions [39]. Normally, *p53* gene is located in the nuclear [40]. It translocates to cytosol and binds to its cytosolic MDM2 after translation, and this binding inhibits *p53* activation. Upon stress signal, *p53* is phosphorylated at serine 15 and releases from MDM2 (mouse double minute 2 homolog) [41], and then activates its downstream factors such as *p21*, BAX (Bcl-2 associated X protein), PUMA (*p53* upregulated modulator of apoptosis), NOXA (phorbol-12-myristate-13-acetate-induced protein 1) or PTEN (phosphatase and tensin homolog) to perform its function.

GLS2 has been proved to be a target of *p53* [42,43]. By up-regulating GLS2 expression (Figure 1), *p53* increases GSH levels and reduces ROS levels, which then inhibits tumorigenesis. Unfortunately, *p53* is mutated in many cancers, which indicates loss of functions. Apart from working on GLS2, *p53* is recently reported to repress expression of SLC7A11 (Figure 1), a key component of the cysteine/glutamate antiporter [44]. SLC7A11 mediates exchange of extracellular cysteine to intracellular glutamate [45], and is overexpressed in several human cancers [44]. Also, *p53* can repress GLUT1 (glucose transporters) and GLUT4, and inhibits PI3K (phosphatidylinositol-3 kinase)–AKT (protein kinase B) and mTOR pathways. These effects of *p53* result in cell growth repression, and then reverse the cancer phenotype [46]. Based on its importantly inhibitive role in cancers, it is particularly interesting in trying to restore or increase *p53* activity in *p53* mutated or loss of function cancers.

#### 2.3.3. Ras in Glutamine Metabolism in Cancers

Oncogenic Ras proteins are identified in 25% of human cancers and are correlated to metabolic alterations. Ras increases utilization of the carbon backbone and amino-nitrogen moieties of glutamine, and promotes glucose consumption [47]. Ras-driven cancers are able to satisfy their nutritional needs through activation of fluid-phase endocytic nutrient uptake, and promotes angiogenesis to increase the tumor blood supply [48]. It has been revealed that glutamine is the major carbon source for the TCA cycle when Ras is activated [49]. The reprogramming of glutamine metabolism is mediated by oncogenic K-Ras in human pancreatic ductal adenocarcinoma [50]. K-Ras can repress glutamate dehydrogenase expression and increase aspartate transaminase expression [50]. At the same time, glutamine depletion can induce proliferation arrest of *K-Ras-*transformed cells [51].

#### 2.3.4. Hypoxia-Inducible Factor (HIF) in Glutamine Metabolism in Cancers

HIF-1, a heterodimer, is composed of HIF-1α and HIF-1β subunits. The expression and activity of the HIF-1α subunit are tightly regulated by cellular oxygen concentration. The expression levels of α subunit are increased during hypoxia whereas the β subunit is constitutively expressed [52]. HIF-1 plays a key role in reprogramming cancer metabolism by activating transcription of many genes that encode glucose transporters as well as glycolytic enzymes, and it also promotes angiogenesis [52,53]. HIF-1 is both necessary and sufficient for reducing mitochondrial oxygen consumption in hypoxia by inducing PDK1 (pyruvate dehydrogenase kinase 1) [54]. In this way, cancers can survival under hypoxia. However, if PDK1 activity is inhibited, continued mitochondrial respiration and resulting oxidative stress will induce cell death.

Solid tumors are often poorly vascularized and contain region hypoxia [55]. This special tumor microenvironment enhances HIF-1α over-expression in the majority of human cancers and their metastases [52], where HIF-1α induces gene expression to promote survival [56]. Under hypoxia, reduced F1Fo ATPase activity can increase phosphate concentration in mitochondria [57]. Increased phosphate promotes GAC (elongated kidney glutaminase variant)-based glutaminase activity, which enhances glutaminolysis to provide increased metabolic and biosynthetic needs for conferring selective advantage to malignant cells [57]. The above-mentioned metabolic regulation makes malignant cells more susceptible to GLS inhibitor and hence can be targeted for cancer therapy [55]. During hypoxia, there is a decrease in glucose-derived citrate due to decreased pyruvate dehydrogenase activity, and an increase in α-KG levels caused by reduced α-KGDH (ketoglutarate dehydrogenase) activity [58]. These changes drive the reverse reaction at IDH (isocitrate dehydrogenase) [58] and then increase citrate production, and finally make cells rely on reductive glutamine metabolism in the lipid synthesis [59]. LKB1 (liver kinase B 1) is a serine/threonine kinase and is often inactivated in human cancer. It has been demonstrated that loss of LKB1 makes cancer cells rely on HIF-1α in the ATP supply, which induces an increase in glycolysis and glutamine consumption [60]. Reasonably, knockdown of HIF-1α in cells without LKB1 promotes a decrease in glutamine consumption by these cells [60].

## 3. Targeting Glutamine Induces Apoptosis in the Cancer Therapy

The reprogrammed metabolism that supports survival and proliferation of cancer cells is now recognized as a hallmark of cancer [61]. This character makes cancers vulnerable to therapeutic strategies that target metabolism. As one of the key energy sources for many cancers, glutamine metabolism has become beyond all doubt a hot topic in cancer therapy in recent years.

Resisting cell death is another hallmark of cancer cells [61]. Cancer cells have several strategies for evading apoptosis, e.g., elimination of *p53* function, an increase in expression of anti-apoptotic factors, down-regulation of pro-apoptotic regulators, or/and short-circuiting death pathway [61]. Promoting apoptosis has long been a particularly interesting topic in cancer therapy. Thus, many studies have been conducted for reactivating or up-regulating apoptosis in cancers. There are mainly two apoptotic pathways: intrinsic and extrinsic pathways. The intrinsic pathway senses and integrates a variety of intracellular origins while the extrinsic pathway receives and processes extracellular death-induced signals involving Fas, DR3 (death receptor 3), and TNF [61]. Both stimulated pathways will subsequently activate pro-apoptotic Bcl-2 family members and/or caspases to execute apoptosis.

There are several strategies for impacting glutamine metabolism, including glutamine deprivation, restriction of glutamine uptake, reduction of glutamine metabolic enzyme activities, usage of glutamine analogues, and disturbance of glutamine metabolism regulators.

### 3.1. Glutamine Deprivation

Glutamine deprivation induces apoptosis through extrinsic or intrinsic pathways, which is dependent on cell type and cell condition. However, glutamine supplementation induces or inhibits cell death, which depends on cell type through variable pathways [3].

**Figure 3:**
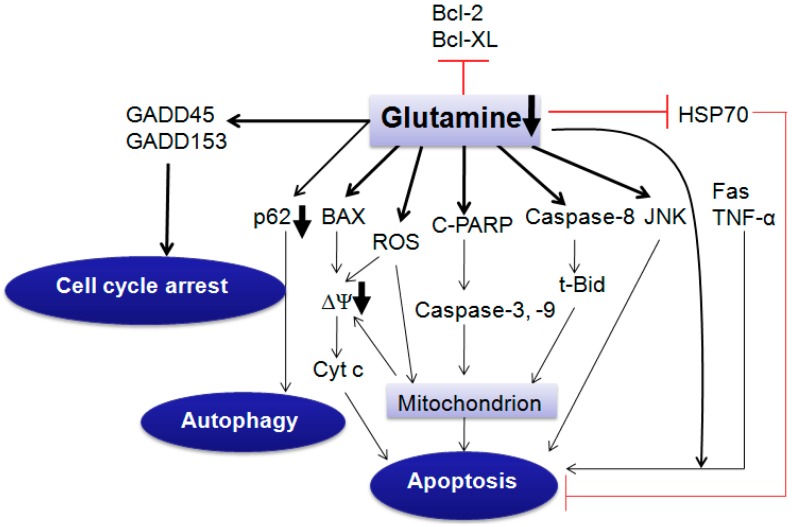
Glutamine deprivation induces cell death or growth arrest. Glutamine deprivation makes cells sensitive to Fas (CD95) ligand, TNF-α and heat shock-mediated apoptosis. Glutamine deprivation induces apoptosis through extrinsic or intrinsic pathway, which is dependent on cell type and cell condition. Cyt c, cytochrome c; C-PARP, cleaved-PARP; t-Bid, truncated Bid; ΔΨ, mitochondrial membrane potential; GADD, growth arrest and DNA damage-induced genes; ROS, reactive oxygen species; JNK, c-Jun N-terminal kinase; HSP70, heat shock protein 70. ┴, inhibiting effect; bold arrow, decreased p62 and ΔΨ after glutamine deprivation.

Glutamine deprivation has been shown to induce apoptosis in hepatoma, hybridoma, leukemia, myeloma and fibroblast cells [3,62,63]. It can stimulate caspase-2, -3 activation and cleaved-PARP expression, and induce cytochrome *c* release (Figure 3). Particularly, the extracellular glutamine level impacts the cell susceptibility to different apoptosis triggers, and cells starving glutamine are more sensitive to Fas (CD95) ligand, TNF-α and heat shock-mediated apoptosis (Figure 3) [3]. It is speculated that HSP70 (heat shock protein 70) reduced by glutamine deprivation [64] prevents downstream of caspase-3-like proteases by inhibiting the function of caspase-activated cytosolic phospholipase A2 and the mitochondrial membrane potential ∆Ψm collapse [65]. In Sp2/0 murine hybridoma cells, glutamine deprivation has been also proved to induce obvious apoptosis by releasing cytochrome c and SMAC (second mitochondria-derived activator of caspases), translocating BAX, activating caspase-9 and caspase-3, and cleaving PARP [66]. Importantly, constitutive expression of *C-MYC* contributes to this apoptosis [66]. However, in the liver cancer cell line, glutamine deprivation does not induce caspase-9 or -8 activation, but stimulates caspase-2 activities [67]. Thus, the glutamine deprivation-induced apoptotic pathway shows cell type specificity.

Glutamine deprivation can also induce apoptosis through GSH reduction. GSH is the main antioxidant in cells and is composed of glutamate, cysteine and glycine. Glutamate, as a composition of GSH, is largely derived from glutamine uptake by cells [8]. Glutamate is also necessary for cells to acquire cysteine and this occurs by virtue of the X_c_-antiporter, which exports glutamate and imports cysteine [10]. GSH plays an important role in the development of cancer cell resistance [68]. It neutralizes intracellular free radicals and prevents the cytotoxic effects, and stimulates the efflux of xenobiotics [45,68]. GSH can also recognize foreign materials and conjugate with them that are excreted out of cells and induce drug resistance [68]. Experiment indicates that mitochondrial GSH depletion, and not cytosolic GSH depletion, are critical factors that lead to activation of cell death [69]. Our previous results show that glutamine deprivation increases mitochondrial ROS and decreases GSH levels in TET21N cell line, and that inhibition of ROS production reverses glutamine deprivation-induced apoptosis, which demonstrates the importance of ROS production during apoptosis. This is consistent with the report of Reid *et al.* [70] that NAC (*N*-acetylcysteine) reverses glutamine deprivation-induced cell death. Inhibitors of γ-glutamylcysteine synthetase have been used to deplete GSH as a strategy for increasing the sensitivity of tumors to certain therapeutic interventions because it is a rate limiting enzyme in GSH synthesis [71].

In addition to inducing apoptosis, glutamine deprivation is also reported to cause autophagy [72] and cell cycle arrest in cancer cells. In HeLa, HCT-116, A549, PC3 and DU145 cells, glutamine deprivation can increase autophagic activity by enhancing levels of the autophagosome-associated form of LC3 (LC3-II) and decreasing autophagy-degraded protein p62 [72,73]. Besides, glutamine deprivation also elevates GADD45 (growth arrest and DNA damage-induced genes) and GADD153 mRNA expression levels within 1.5 h in subconfluent cultures of the human breast cell lines HBL100 and TSE (Figure 3). It is suggested that the expression of GADD genes contributes to growth arrest and/or protection from metabolic damage during glutamine-poor conditions [74]. Interestingly, glutamine deprivation leads to the up-regulation of the monocarboxylate transporter 1, which causes a higher sensitivity of cancer cells to 3-bromopyruvate (an antitumor agent under clinical development) both *in vivo* and *in vitro* [72,75]*.* This indicates potential ability of targeting glutamine metabolism to be synergistic with other chemotherapy drugs.

### 3.2. Restriction of Glutamine Uptake

As previously mentioned, increased glutamine transporters are also accounted for glutamine addiction in most cancer cells. So glutamine transport inhibition is another way to restrict glutamine metabolism.

ASCT2 and LAT1 are up-regulated in a variety of cancerous tissue [18,76]. Some ASCT2 inhibitors including tamoxifen, raloxifene and GPNA (γ-l-glutamylp-nitroanilide) have been shown to be effective in bench research (Table 1) [2,77]. Inhibition of ASCT2 function by chemicals or shRNA *in vitro* decreases glutamine uptake, cell cycle progression through down-regulation of E2F transcription factors and mTORC1 pathway in LNCaP (lymph node carcinoma of prostate) and PC-3 prostate cancer cell lines, and in PC-3 cell xenografts [78]. Also, ASCT2 directly participates in cell survival signaling, and plays a major role in driving the glutamine-dependent growth of the rat astrocytoma-derived C6 cell line [20] and human neuroblastoma cell line SK-N-SH [21]. Inhibition of LAT1 caused by BCH (2-aminobicyclo-(2,2,1)-heptane-2-carboxylic acid) in H1395 lung cancer cell line and KKU-M213 (cholangiocarcinoma cells derived from Thai patients with intrahepatic cholangiocarcinoma)cells can reduce cellular l-leucine uptake, and consequently inhibits mTOR pathway activity, which finally reduces cell proliferation and viability [79,80]. In addition to inhibition of glutamine access by blocking transporters, asparaginase treatment can also prevent glutamine supplementation [81].

Although restriction of glutamine supplementation is regarded as a potent strategy for curing some cancers, it is not universal to all cancers. In the mouse fibrosarcoma cell line L929, glutamine omission desensitizes cells to TNF-α-induced cytotoxicity [82]. In human hepatoma cell (SMMC-7721), cell proliferation is inhibited and cells are induced to apoptosis when glutamine was added to cell culture [83]. Thus, there is specificity of cell type in the inhibition of glutamine metabolism for cancer therapy.

**Table 1:** Compounds targeting glutamine metabolism in cancer research.

Compound	Target	References
BPTES	GLS1	[84,85,86,87,88,89]
968	GAC	[85,90,91,92,93]
CB-839	GLS1	[15,94]
Ebselen	GLS1, GLS2	[95]
Chelerythrine	GLS1, GLS2	[95]
Apomorphine	GLS1, GLS2	[95]
DON	Glutamine antagonist	[96,97,98,99]
Acivicin	γ-Glutamyl transpeptidase glutamine amidotransferase; Glutamine antagonist	[99,100,101,102]
BCH	Glutamine transporter (SLC7A5)	[79,80]
α-Methyl-dl-tryptophan	Glutamine transporter (SLC6A14)	[103,104]
Tamoxifen	Glutamine transporter (ASCT2)	[77]
Raloxifene	Glutamine transporter (ASCT2)	[77]
GPNA	Glutamine transporter (ASCT2)	[2,77]
EGCG	GDH	[105,106,107]
l-Asparaginase	Glutamine	[81,108,109]
Phenylacetate	Glutamine	[110,111]

BPTES, bis-2-(5-phenylacetamido-1,2,4-thiadiazol-2-yl)ethyl sulfide 3; 968, 5-(3-bromo-4-(dimethylamino) phenyl)-2,2-dimethyl-2,3,5,6-tetrahydrobenzo[a]phenanthridin-4(1H)-one; CB-839, *N*-(5-(4-(6-((2-(3-(trifluoromethoxy)phenyl)acetyl)amino)-3-pyridazinyl)butyl)-1,3,4-thiadiazol-2-yl)-2-pyridineacetamide; DON, 6-diazo-5-oxo-l-norleucine; BCH, 2-aminobicyclo-(2,2,1)-heptane-2-carboxylic acid; GPNA, γ-l-glutamylp-nitroanilide; EGCG, epigallocatechin gallate; GLS, Glutaminase; GAC, Enlongated kidney glutaminase variant; GDH, Ketoglutarate dehydrogenase.

### 3.3. Inhibition of Glutaminase

Due to the enhanced glutamine metabolism in most cancers, GLS is being explored as a significant agent in therapeutic interventions. It is also proposed as a biomarker of glutamine-dependence and therapeutic target [81]. BPTES (bis-2-(5-phenylacetamido-1,2,4-thiadiazol-2-yl)ethyl sulfide 3) and 968 (5-(3-bromo-4-(dimethylamino)phenyl)-2,2-dimethyl-2,3,5,6-tetrahydrobenzo[a]phenanthridin-4(1H)-one) have been proved to be the most frequent GLS inhibitors in recent studies. Several derivations or analogs of BPTES [112] and a newly discovered glutaminase 1 inhibitor CB-839 are also being researched. The glutamine antagonist such as DON (6-diazo-5-oxo-l-norleucine) found previously is nonspecific, has toxic or side effects, and inhibits several enzymes of glutamine utilization [96]. Drugs such as ebselen, chelerythrine and apomorphine were also regarded as GLS inhibitors in a recent research (Table 1) [95].

#### 3.3.1. Glutaminase (GLS)

Mammals express three isoforms of GLS including GLS1 (KGA (kidney GA isoform), GAC) and GLS2 (liver GA isoform, or LGA (liver glutaminase) [57,113]). KGA and GAC are two isoforms of the same gene kidney-type GLS in chromosome 2, and they have the same N-terminal but distinct C-terminal [84]. The KGA isoform is highly expressed in kidney, brain, intestine, and cells of the immune system but not in liver, whereas GAC is expressed in heart, pancreas, placenta, lung and in many transformed cells but not in brain and liver [84,114]. GLS2 is primarily found in adult liver [115]. The new evidence has revealed that GLS2 expression also occurs in extrahepatic tissues, such as brains [43]. At cellular level, GLS2 is an inner mitochondrial membrane enzyme [43], and KGA and GAC most likely localize in the mitochondria [85].

GLS1 maintains acid-base balance during metabolic acidosis in the kidney and generates glutamate that in turn acts as an excitatory neurotransmitter in the central nervous system [86]. In intestinal epithelium, KGA initiates the catabolism of glutamine, which serves as a major respiratory fuel source [114]. However, GLS2 most likely works as enzyme to catalyze glutamine metabolism, and then increases cellular levels of GSH and NADH, and decreases ROS levels in cells. Thus, GLS2 is regarded as a tumor suppressor [116].

Among three GLS, e.g., GAC, LGA, KGA, KGA or/and GAC is most expressed in many cancers and promotes caner proliferation. KGA is up-regulated in tumors from diverse organs and cells including breast, lung, liver, brain and B cells [55,85,90,117,118,119], while GAC is predominantly expressed in human breast cancer cell line that exhibits a high rate of glutamine utilization and GLS activity [114]. In contrast, GLS2 expression is reduced in human liver tumors when compared to normal tissues [120], which may be one of the reasons for tumorigenesis. At the same time, GLS2 over-expression reduces the formation of cancer cell colonies [52].

#### 3.3.2. Regulators of GLS

GLS expression and activation are regulated by several factors. GLS1 is activated by high phosphate levels (allosteric activation) and strongly inhibited by the end-product glutamate, whereas GLS2 is activated by low phosphate levels and not inhibited by glutamate [121]. As one of the end products of glutaminolysis, ammonia is an inhibitor of KGA and activator of LGA. However, ammonia is an activator of KGA at high concentrations [122].

EGF (epidermal Growth Factor) and oncogenes also participate in GLS regulation. KGA activity is activated by EGF via the Raf-Mek-Erk signaling module [87]. *C-MYC* induces GLS1 expression and promotes oncogenic transformation and cancer cell proliferation (Figure 2) [8]. *C-MYC* transcriptionally represses microRNAs 23a and 23b that in turn suppress transcription of the GLS1 gene, which results in greater expression of mitochondrial GLS in human P-493 B lymphoma cells and PC3 prostate cancer cells [31]. Another GLS1 inhibitor is NF-Κb (nuclear factor kappa-light-chain-enhancer of activated B cells), which is able to inhibit miR-23a and miR-23b [90]. GLS2 is confirmed to be a *p53* target gene both in normal and tumor cells (Figure 2) [43] because human GLS2 gene promoter contains a *p53* consensus DNA-binding element [52]. Besides *p53*, TAp73 (a member of *p53* family) also drives the expression of GLS2 in neuroblastoma cells [123]. In a recent study, ErbB2 can also activate GLS1 in both protein and mRNA level in breast tumor cells via activations of NF-κB and MAPK (mitogen-activated protein kinase) pathways because inhibition of NF-κB and MAPK reduces GLS1 expression [124]. In addition, retinoblastoma protein is also involved in GLS1 mRNA and protein expression, and depletion of retinoblastoma protein in mouse embryonic fibroblasts increases GLS1 activity [125]. Interestingly, STAT1 (signal transducer and activator of transcription 1) at phosphorylation status is found to directly bind to the GLS1 promoter in monocyte-derived-macrophages, which is involved in IFN-α. IFN-α treatment that can increase STAT1 phosphorylation and GLS mRNA and protein expression levels [126].

#### 3.3.3. GLS1 Inhibitors in Cancer Therapy

##### BPTES (Bis-2-(5-phenylacetamido-1,2,4-thiadiazol-2-yl)ethyl sulfide 3)

BPTES is a specific GLS1 inhibitor [84,86], and inhibits GLS1 in an uncompetitive manner by facilitating the formation of an inactive tetramer (Table 1) [84,88]. BPTES binds to KGA at the dimeric interface near the active site and triggers a dramatic conformational change of the key loop (Glu312-Pro329) near the catalytic site [87,89]. It is fully effective against GLS activity regardless of whether BPTES is added to GLS before or after the allosteric activation of GLS by inorganic phosphate [85]. In addition, BPTES treatment also represses glutamine uptake [127], elevates ROS levels and reduces GSH level [55]. However, BPTES has high molecular weight (534), poor solubility and low bioavailability, which makes it an imperfect candidate for GLS inhibition, even though the persistent use of BPTES during glutamine metabolism research was found to be acceptable due to its low adverse effects and high efficiency.

BPTES treatment prolongs the survival of animals with liver tumorigenesis without obvious side effects, causing a delay in G1-S cell-cycle transition and subsequent DNA replication arrest, cell death, and fragmentation [120]. BPTES also suppresses the growth of acute myeloid leukemia cells that express mutant IDH when compared with those that express wild type IDH [128]. Instead of catalyzing isocitrate and NADP^+^ to α-KG and NADPH, mutated IDH1 converts α-KG to d-2-hydroxyglutarate. This selectively inhibitive ability of BPTES in tumors implicates a potential therapeutic strategy [119]. Intriguingly, BPTES reduces the conversion of [1-13C]-pyruvate to alanine but not to lactate [129], which is associated with elevated glycolytic intermediates that may reflect a compensatory increase in glycolysis to produce α-KG and to maintain homeostasis [12,32,56,119]. As previously mentioned, *MYC* is involved in glutamine addiction in many cancer cells while the inhibitive ability of BPTES is influenced by the *MYC* status in some cases. BPTES induces apoptosis in IMR90-ERMYC and HA1E-MYCER cells via a *MYC*-dependent manner because cells without *MYC* show less degree of apoptosis [32]. BPTES also inhibits growth of a *MYC*-dependent human B cell lymphoma cell line (P493) by blocking DNA replication, leading to cell death and fragmentation [120]. Also, BPTES increases heat shock protein 90 (Hsp90) inhibitor, e.g., 17AAG-induced Tsc2^−/−^ MEFs cell death [130], which shows a synergy effect.

##### 968 (5-(3-Bromo-4-(dimethylamino)phenyl)-2,2-dimethyl-2,3,5,6-tetrahydrobenzo[a]phenanthridin-4(1H)-one)

968 is a specific inhibitor of GAC, and acts as an allosteric manner to prevent GAC activation in cells by blocking the posttranslational modification(s) (Table 1) [91]. Unlike BPTES, 968 can only inhibit GAC that is not activated. If 968 is added to GAC just seconds before activation, 968 can exhibit its full inhibitive effect [85]. Also, BPTES induces tetramer formation while 968 binds to the monomeric form of GAC to induce a conformational change that renders the enzyme inactive [92,93]. 968 is firstly found as a oncogenic diffuse B cell lymphoma inhibitor, and is able to inhibit the transforming activity of Rho GTPase, which promotes GLS1 activity by activating NF-κB [90]. In the following researches, the target of 968 has been identified to be GAC [90]. 968 has specific effects on the growth, migration and invasive activity of transformed/cancer cells, and has no effects on the growth or morphology of their non-transformed cellular counterparts [91,93]. Growth of breast, brain and pancreatic cancer cells has been reported to be inhibited by 968 [131].

968 treatments have been shown to induce cytotoxicity in multiple human breast cancer cell lines, accompanying by down-regulating anti-apoptotic and/or metastasis genes including AKT, Bcl-2 (B-cell lymphoma-2) and *MYC* [132]. These changes of gene expression result in activation of apoptosis, and decrease invasiveness [132]. More importantly, 968 increases MDA-MB-231 sensitivity to chemotherapy drug such as doxorubicin [132], and also sensitizes EGFR activating mutation-expression GBM cell lines to mTOR-targeted therapy through down-regulating α-KG both *in vitro* and *in vivo* [133,134]. 968 significantly decrease the uptake of glutamine and secretion of by-products of glutamine catabolism such as ammonia, and suppress GBM cell proliferation in a dose-dependent manner [134]. Interestingly, with activated mTORC1 signaling, combination of 968 and inhibitor of Hsp90, e.g., 17AAG increases apoptosis induced by 17AAG alone in Tsc2^−/−^ MEFs [130]. Simultaneous inhibition of GLS1 by 968 and tissue transglutaminase by inhibitor e.g., monodansylca-daverine, result in a synthetic lethality across a panel of assorted cancer cell lines [131]. The above-mentioned studies demonstrate that 968 is effective in not only inhibiting cancer cell growth, but also increasing cancer cells’ sensitivity to other chemotherapy drugs or other metabolic signaling inhibitors.

In addition to directly inhibiting glutamine metabolism, 968 also restrain other metabolite production [134] and carcinogenesis. 2-HG (hydroxyglutarate) is a metabolite of IDH1 mutation, and can lead to oxidative stress [119]. 968 treatment reduces intracellular 2-HG level by 80%–90% in breast tumor cell lines [135]. ESVs are extracellular shed vesicles, and are considered as cancer-specific signatures associated with dysregulated glutamine metabolism [136]. 968 significantly reduce the total ESVs in cancerous epithelial cell lines and inhibit tumorigenesis [136].

##### CB-839 (*N*-(5-(4-(6-((2-(3-(Trifluoromethoxy)phenyl)acetyl)amino)-3-pyridazinyl)butyl)-1,3,4-thiadiazol-2-yl)-2-pyridineacetamide)

CB-839 is another potent and selective GLS1 inhibitor. It has more potent inhibition on GLS1 than BPTES. It is a noncompetitive inhibitor and displays potency that is independent on glutamine concentration and exhibits time-dependent and slowly reversible kinetics (Table 1) [15]. It has been proved to inhibit proliferation of triple-negative breast cancer cell lines which are highly resistant to chemotherapy [15], and decrease viable cell number in leukemia and induce apoptosis [94]. Also, it inhibits the growth of lymphoma, myeloma, mesothelioma, acute leukemias and non*-*small cell lung cancer [137].

Glutamate, a-KG, aspartate, fumarate and malate are reduced during CB-839 treatment, accompanying by decreased respiratory capacity [15,94]. More interestingly, in THP1 cell lines stably transduced with doxycycline-induced mutant IDH1-R132H or IDH2-R140Q construct, CB-839 treatment can increase CD11b (differentiation marker) levels and morphological signs of differentiation, and reduce intracellular 2-HG levels [94], which indicates a more potent antitumor ability. CB-839 treatment also significantly decreases the viability of myeloma cells without markedly impacting peripheral blood mononuclear cells (PBMCs) from normal healthy donors, which suggests specific anti-myeloma activity and a favorable therapeutic index for CB-839 [138]. Both autophagy and the caspase-mediated apoptotic pathway contribute to anti-myeloma activity of CB-839 [138]. Recently, CB-839 has been shown to synergistically sensitize leukemic cells to priming with the Bcl-2 inhibitor, e.g., ABT-199 [139].

#### 3.3.4. GLS2 in Cancer Therapy

In contrast to GLS1, GLS2 is widely accepted as tumor suppressor and inhibits tumor formation via ectopic expression. However, GLS2 has been shown to play an important role in radioresistance of cervical cancer patients in a recent study [140]. Knock-down of GLS2 increases the intracellular ROS levels, and substantially enhances radiosensitivity of cervical cancer [140]. Lee *et al.* have found that a series of alkyl benzoquinones preferentially inhibit GLS2 rather than GLS1 [141]. Treatment with the alkyl benzoquinones decreases intracellular GLS activity, glutamate levels, carcinoma cell proliferation and anchorage-independent colony formation, and induces autophagy via AMPK (AMP-activated protein kinase)-mediated mTORC1 inhibition [141].

## 4. Conclusions

Dysregulated cancer metabolism promotes cell proliferation because of increased energy production and metabolite synthesis, and decreases drug-induced apoptosis, and confers therapeutic resistance [56]. Thus, targeting cancer cell metabolism becomes a novel way to address this challenge.

Glutamine is a versatile amino acid and is used to support cell growth and proliferation. It has been proved that glutamine is irreplaceable especially for most tumor cells. Restriction of glutamine metabolism through depriving glutamine, blocking glutamine transporters or inhibiting GLS activity have been proved to be effective in inhibiting tumor cell growth by inducing apoptosis and/or autophagy both *in vitro* and *in vivo*. Especially, these newly discovered GLS inhibitors (968 and CB-839) have high efficiency and low adverse effects. Besides, they also are effective in increasing cancer cells’ sensitivity to other common chemotherapy when they work together. However, it is not advised to inhibit glutamate production in the central nervous system because glutamate is an important neurotransmitter. Thus, inhibitors that cannot cross the blood–brain barrier may be useful in non-central nervous system cancer [142].

## 5. Outlook

As the field of tumor therapy develops, problems become more obvious. As previously mentioned, cancer cells are highly heterogeneous, even the same type of cancers have different metabolic mechanisms, and molecular and clinical characteristics. Thus, it is of great value to explore the common properties of the homologous cancers. Glutamine metabolism restriction and chemotherapy drugs together are also feasible since targeting metabolic adaptations of cancer cells has the potential to sensitize cancer cells to anticancer drugs [72]. Besides, more studies are required to figure out the molecular mechanism in the relationship between glutamine and apoptosis in cancers. In some cancers, glucose and glutamine are complementary to each other. Inhibiting one nutrient may be useless in treating some cancer cells when another nutrient is available. A possible solution to this is to combine inhibitors of glutamine and glucose, which needs further consideration and studies. Disconnection of preclinical and clinical research is another big problem. Two dimensional cell cultures have shown their limitations, and the tumor microenvironment is becoming increasingly important for developing cancer therapy; thus three-dimensional cell culture methods and more clinical data are required. More effective and less toxic analogous of glutamine must also be identified since the existing glutamine mimics are systemically toxic and there is inexplicable variability among increasing numbers of cancers [81].

## Abbreviations

17AGG	17-Allylaminogeldanamycin
968	5-(3-Bromo-4-(dimethylamino)phenyl)-2,2-dimethyl-2,3,5,6-tetrahydrobenzo[a]phenanthridin-4(1H)-one
α-KG	α-Ketoglutarate
ABT-199	Venetoclax
ALL	Acute Leukemias
Akt	Protein kinase B
AMPK	AMP-activated protein kinase
ATP	Adenosine-triphosphate
ASCT	System ASC amino acid transporters 1 and 2
BAX	Bcl-2 associated X protein
BCH	2-Aminobicyclo-(2,2,1)-heptane-2-carboxylic acid
BPTES	Bis-2-(5-phenylacetamido-1,2,4-thiadiazol-2-yl)ethyl sulfide 3
CB-839	*N*-(5-(4-(6-((2-(3-(Trifluoromethoxy)phenyl)acetyl)amino)-3-pyridazinyl)butyl)-1,3,4-thiadiazol-2-yl)-2-pyridineacetamide
CD95	Cluster of differentiation 95
Dbl	Diffuse B cell lymphoma
DON	6-Diazo-5-oxo-l-norleucine
DR	Death receptor
DMAP1	Dnmt1 associated protein
EAA	Essential amino acid
EGF	Epidermal Growth Factor
ErbB2	Receptor tyrosine-protein kinase erbB-2
GAC	Enlongated kidney glutaminase variant
GADD	Growth arrest and DNA damage-induced genes
GBM	Glioblastoma multiforme
GCL	Glutamate-cysteine ligase
GCS	Glutamylcysteine synthetase
GLS	Glutaminase
GLUD	Glutamate dehydrogenase
GLUT	Glucose transporters
GOT	Aspartate transaminase
GPNA	γ-l-Glutamylp-nitroanilide
GSH	Glutathione
HG	Hydroxyglutarate
HIF	Hypoxia-inducible factor
HSP	Heat shock protein
IDH	Isocitrate dehydrogenase
KGA	Kidney glutaminase
KGDH	Ketoglutarate dehydrogenase
LAT	l-Type amino acid transporters
LC3	Microtubule-associated protein 1A/1B-light chain 3
LDHA	Lactate dehydrogenase
LGA	Liver glutaminase
LKB	Liver kinase B
MAPK	Mitogen-activated protein kinase
MDM2	Mouse double minute 2 homolog
mTOR	Mammalian target of rapamycin
NAC	*N*-Acetylcysteine
NADPH	Nicotinamide adenine dinucleotide phosphate
NF-κB	Nuclear factor kappa-light-chain-enhancer of activated B cells
NOXA	Phorbol-12-myristate-13-acetate-induced protein 1
OAA	Oxaloacetic acid
PARP	Poly(ADP-ribose) polymerase
PC	Pyruvate carboxylase
PDK	Pyruvate dehydrogenase kinase
PDH	Pyruvate dehydrogenase
PI3K	Phosphatidylinositol-3 kinase
PP242	2-(4-Amino-1-isopropyl-1H-pyrazolo[3,4-d]pyrimidin-3-yl)-1H-indol-5-ol
PTEN	Phosphatase and tensin homolog
PUMA	p53 upregulated modulator of apoptosis
ROS	Reactive oxygen species
SLC7A5	Solute carrier family 7 member 5
SMAC	Second mitochondria-derived activator of caspases
SN1	System N amino acid transporter 1
STAT	Signal transducer and activator of transcription 1
TCA	Tricarboxylic acid cycle
TNF-α	Tumor necrosis factor-α
